# Plant defense: ARR11 response regulator as a potential player in Arabidopsis

**DOI:** 10.3389/fpls.2022.995178

**Published:** 2022-09-21

**Authors:** Gaia Salvatore Falconieri, Laura Bertini, Elisabetta Bizzarri, Silvia Proietti, Carla Caruso

**Affiliations:** Department of Ecological and Biological Sciences, University of Tuscia, Viterbo, Italy

**Keywords:** ARR11, plant hormones, Arabidopsis, plant defense, necrotrophic pathogen

## Abstract

Plant growth and response to environmental cues are largely driven by hormones. Salicylic acid (SA)- and jasmonic acid (JA)-mediated defenses have been shown to be effective against different types of attackers. SA-mediated defense is mainly effective against biotrophic pathogens and phloem-feeding insects, whereas JA-mediated defense is effective against necrotrophic pathogens and tissue-damaging insects. Cytokinins (CKs) are classic growth hormones that have also emerged as plant immunity modulators. Evidence pointed out that CKs contribute to the defense responses mediated by SA and JA, acting as hormone modulators of the SA/JA signaling backbone. Recently, we identified in Arabidopsis a type-B response regulator 11 (ARR 11) involved in cytokinin-mediated responses as a novel regulator of the SA/JA cross-talk. Here we investigated plant fitness and resistance against the fungal necrotrophic pathogen *Botrytis cinerea* in Arabidopsis wild-type Col-8 and defective *arr11* mutant following SA, JA, CK single or combined treatment. Our results demonstrated that the CK and SA/JA/CK combination has a positive outcome on plant fitness in both Arabidopsis Col-8 and *arr11* mutant,. The triple hormone treatment is efficient in increasing resistance to *B. cinerea* in Col-8 and this effect is stronger in *arr11* mutant. The results will provide not only new background knowledge, corroborating the role of ARR11 in plant-defense related processes, but also new potential opportunities for alternative ways of protecting plants from fungal diseases.

## Introduction

Plant hormones are key players in plant immunity. The type of attacker can determine what type of hormone accumulates in the plant, and each hormone regulates its own core pathway in the immune network (Aerts et al., 2020; Aerts et al., 2022). The defense pathways regulated by jasmonic acid (JA) and salicylic acid (SA), which form the backbone of the hormone‐regulated part of the immune system, are among the most studied (Wasternack and Song, 2017; Zhang and Li, 2019; Aerts et al., 2020). The JA pathway can be subdivided into two branches. The ERF branch is activated by necrotrophic pathogen infection while the MYC branch generally provides protection against chewing insects. The SA pathway is considered primarily directed against biotrophic pathogens (Pieterse et al., 2012). Additionally, other plant hormones, such as auxins, abscisic acid (ABA), cytokinins (CKs), gibberellins, and brassinosteroids, which have been thoroughly described to regulate plant development and growth, have emerged as key regulators of plant immunity (Pieterse et al., 2012). In particular, CKs are among the most important signaling molecules for regulating growth and development throughout the life of the plant and largely involved in cell division, growth and organogenesis, vascular differentiation, lateral root initiation, gravitropism and phototropism as well as fruit development (Osugi and Sakakibara, 2015; Wu et al., 2021). All plant hormonal pathways are linked to each other in a huge, complex, and largely still obscure network, to balance the response to developmental and environmental cues, as a cost-saving strategy (Pieterse et al., 2012; Thaler et al., 2012; Aerts et al., 2020). The classical example of cross-talk in defense regulation is that between the SA and JA pathways. The antagonism between these two pathways is the most studied and prevalent, although large-scale additive and synergistic interactions have been reported (Hickman et al., 2019; Aerts et al., 2020). The synergistic or antagonistic impact of CKs on SA- and JA-mediated defense responses has been reported in recent years (Zhang et al., 2022 and references therein). For instance, dose-dependent CKs application can modulate SA-mediated resistance to the biotrophic oomycete pathogen *Hyaloperonospora arabidopsidis* (Argueso et al., 2012). Moreover, the synergistic interaction between SA and CK has been shown to increase rice resistance to infection of the blast fungus *Magnaporthe oryzae* (Jiang et al., 2013). In addition, CKs have been reported to enhance JA-mediated resistance to the virulent necrotrophic fungus *Alternaria brassicicola* (Choi et al., 2010). To the best of our knowledge, beyond the combination CK/SA and CK/JA, the impact of SA/JA/CK combined treatment on pathogen resistance, as well as on growth, has not yet been investigated. Recently, from a GWA study performed in Arabidopsis, we identified ARR11 as a novel player involved in the effect of SA on JA pathway, using *PDF1.2* gene as JA-marker. Additionally, in *arr11* defective mutant we found altered resistance against the fungal necrotrophic pathogen *Botrytis cinerea*, compared to the wild-type Col-8 (Proietti et al., 2018). Furthermore, besides the involvement of ARR11 in SA/JA cross-talk, this protein has been shown to play a role in cross-communication between CK and ABA signaling (Huang et al., 2018). ARR11 (TAIR code: At1g16770) belongs to the family of type-B ARRs (Arabidopsis Response Regulators) which plays a pivotal role in the early transcriptional response of plants to cytokinins (Kushwah et al., 2011). The type-B ARRs are structurally related, each possessing a receiver domain phosphorylated on a conserved Asp residue, as well as a long C-terminal extension with a Myb-like DNA-binding domain (Imamura et al., 1999; Hosoda et al., 2002). The ability of the Myb-like domain to bind DNA has been demonstrated in several studies (Sakai et al., 2000; Hosoda et al., 2002) and multiple lines of evidence support a role of type-B ARRs as transcription factors (Liang et al., 2012; Tsai et al., 2012).

Here we investigated the involvement of ARR11 in SA/JA/CK-mediated responses in Arabidopsis. We demonstrated that SA/JA/CK triple treatment has a positive impact on plant fitness and resistance against *B. cinerea* in both Arabidopsis wild-type Col-8 and *arr11* mutant. Nevertheless, in the latter, the combined hormone treatment is even more efficient in increasing resistance to *B. cinerea* suggesting ARR11 as a player in plant defense related processes.

## Materials and methods

### Plant material and growth conditions

*A. thaliana* T-DNA line in Col-8 background (*arr11*) was acquired from NASC (http://arabidopsis.info/) (SALK_006544C), as already described (Proietti et al., 2018). Seeds of the Arabidopsis *arr11* and Col-8 were sown in cultivation containers filled with autoclaved river sand, supplied with half-strength Hoagland solution (Sigma, Steinheim, Germany). To reach high relative humidity for germination, the cultivation containers were enclosed in a tray with water and covered with a transparent lid. Seeds were incubated for 2 days at 4°C in the dark to ensure homogeneous germination, after which the tray was moved to a growth chamber with 8-h day/16-h night rhythm, a temperature of 21°C, and a light intensity of 100 μmol m^-2^ sec^-1^. After a week, the lids were slightly opened and gradually removed over a 2-day period. Ten-day-old seedlings were transplanted to individual pots containing autoclaved mixture of river sand and potting soil (1:1, v:v). Pots were supplied with water from the bottom three times per week. At the age of 3 weeks the plants were supplied once a week with half strength Hoagland solution.

### Chemical treatments

Five-week-old *A. thaliana* plants (Col-8 and *arr11* T-DNA line) were treated individually with SA (Sigma, Steinheim, Germany), MeJA (Serva, Brunschwig Chemie, Amsterdam, the Netherlands), CK (Sigma, Steinheim, Germany) or in combination, by dipping plants in a solution containing 0.015% (v:v) Silwet L77 (Van Meeuwen Chemicals BV, Weesp, the Netherlands) supplemented with either 1 mM SA or 100 μM MeJA or 15 μM CK or a combination of the three hormones at the same concentration. MeJA was diluted from a 1000-fold concentrated stock in 96% ethanol. The mock solution contained 0.015% Silwet L77 only. The 5^th^ and 11^th^ leaves from 3 plants were harvested 24 hours after treatment, immediately frozen in liquid nitrogen and then stored at -80°C before further analysis.

### Plant fitness parameters

Leaf (5^th^ and 11^th^) area was measured by using a ruler. Leaf dry weight was measured on a microbalance (0.001 g resolution) after drying the leaves in an oven at 60°C. Flowering time was determined as the time of first flower appearance after treatment. To define seed production, plants were watered every other day until they stopped producing new flowers. Inflorescences were harvested when plants had finished flowering and the seeds were weighed on a microbalance with a 0.0001 g resolution.

### RNA extraction and RT-qPCR

Total RNA was isolated as described in Oñate-Sánchez and Vicente-Carbajosa, 2008. DNase treatment was performed by using DNase I (Fermentas) at the concentration of 0.5U/μg RNA. To convert DNA-free total RNA into cDNA, RevertAid H minus Reverse Transcriptase (Fermentas) was used. PCR reactions were made in optical 96-well plates (Applied Biosystems) with ABI PRISM^®^ 7900 HT sequence detection system by using SYBR^®^ Green to detect the synthesis of double-stranded DNA. The following thermal profile was used: 50°C for 2 min, 95°C for 10 min, 40 cycles of 95°C for 15 s and 60°C for 1 min. Amplicon dissociation curves were monitored after cycle 40 by heating from 60 to 95°C with a ramp speed of 1°C/min. Transcript levels were calculated relative to the reference gene PP2AA3 (Czechowski et al., 2005) using the 2^-ΔΔCT^ method previously described (Livak and Schmittgen, 2001). The AGI numbers of the studied genes are At1g67710 (ARR11), At5g44420 (PDF1.2), and At1g13320 (PP2AA3). Fold change was calculated relative to the mock treatment. A one-way and two-way ANOVA was performed on fold changes to determine the statistical significance of differences in expression levels of *ARR11* and *PDF1.2*, respectively

### Pathogen bioassay

*Botrytis cinerea* bioassay has been performed according to Van Wees et al. (2013), with some modification. *Botrytis cinerea* strain B05.10 was grown for 2 weeks on half-strength potato dextrose agar (PDA; Difco Laboratories, Leeuwarden, the Netherlands) plates containing penicillin (100 µg mL^-1^) and streptomycin (200 µg mL^-1^) at room temperature. *B. cinerea* spores were collected, filtered through glass wool, and re-suspended in half-strength potato dextrose broth (PDB; Difco Laboratories, Leeuwarden, the Netherlands) to a final density of 1x10^5^ spores mL^-1^. After a 3-h incubation period, 5-week-old plants were inoculated by applying 5µl droplets of the spore suspension to six leaves of each plant. Plants were infected after 24 hrs by hormone/mock treatments, performed as in par. 2.2. Plants were placed in a closed box to increase relative humidity to 100% to stimulate the infection. Three days after *B. cinerea* inoculation, the symptoms were scored in five disease severity classes ranging from Stage I, lesion 2 mm; stage II, lesion 2 mm + chlorosis; stage III, lesion 2–4 mm + chlorosis; stage IV, lesion > 4 mm + chlorosis; stage V (lesion > 4-5 mm with tissue maceration). Percentage of leaves in each class was calculated per plant (χ^2^ test; n=24 plants per line).

### ROS detection

ROS detection was performed as described in Proietti et al., 2019, on leaves of Col-8 and *arr11* plants, three days after *B. cinerea* infection, with or without hormone treatment Briefly, ROS production was detected by using 2’,7’-dichlorofluorescein diacetate (DCFH_2_-DA; Sigma Aldrich, St. Louis, MO, USA), that is oxidized to highly fluorescent dichlorofluorescein (DCF) when ROS are present. Two leaves from each of six 5-week-old Col-8 and *arr11* plants were collected. One leaf from each plant was incubated at room temperature in 20 mM DCFH_2_-DA in 10 mM Tris-HCl solution (pH 7.4) for 45 min in the dark. As a negative technical control, the other leaf was incubated in 10 mM Tris-HCl (pH 7.4) only, under the same conditions. After the staining, the samples were washed three times in 10 mM Tris-HCl (pH 7.4) for 10 min to eliminate the excess of fluorophore and finally mounted on glass slides. Fluorescence was then observed under a LSM 710 confocal microscope (Carl Zeiss Microscopy GmbH, Jena, Germany) with Plan Neofluar 20/1.30 objective. Two laser excitations lines were used (i.e., 488 nm for probe detection and 561 nm for chlorophyll auto-fluorescence). Data were processed using Image J software (http://rsbweb.nih.gov/ij/).

### Thiobarbituric acid reactive substance measurement

TBARS levels were used to assess lipid peroxidation following the protocol described in Bertini et al., 2019 and in Proietti et al., 2021, on leaves of Col-8 and *arr11* plants, three days after *B. cinerea* infection, with or without hormone treatment. Briefly, four hundred milligrams of frozen leaves were finely ground using a mortar and pestle under continuous addition of liquid nitrogen. The powder was resuspended in 3 mL of trichloroacetic acid (TCA), 0.1%, and mixed on the vortex until homogenized. Following centrifugation at 13,000 rpm for 10 min, 400 µL of the supernatant (or 0.1% TCA for the blank) was added either to 1 mL of 0.5% TBA in 20% TCA (+TBA solution) or to 1 mL of 20% TCA (–TBA solution) (dilution factor 1:3.5). Samples were then incubated at 80°C for 30 min and cooled on ice. After centrifugation at 13500 rpm for 5 min, the absorbance was measured both at 532 nm, that represents the maximum absorbance of the TBA–TBARS complex, and at 600 nm to allow correction of non-specific turbidity. To calculate the TBARS equivalent (nmol mL^–1^), the ϵ_µM_ (0.155 µM^−1^ cm^−1^) of malondialdehyde (MDA), one of the main products of membrane damage, was used according to the following formula:

[A/εµM MDA] × dilution factor

where A = [(A_532nm_ (+TBAsol) − A_600nm_ (+TBAsol))] − [(A_532nm_ (−TBAsol) − A_600nm_ (−TBAsol)]

## Results

**Figure 1:**
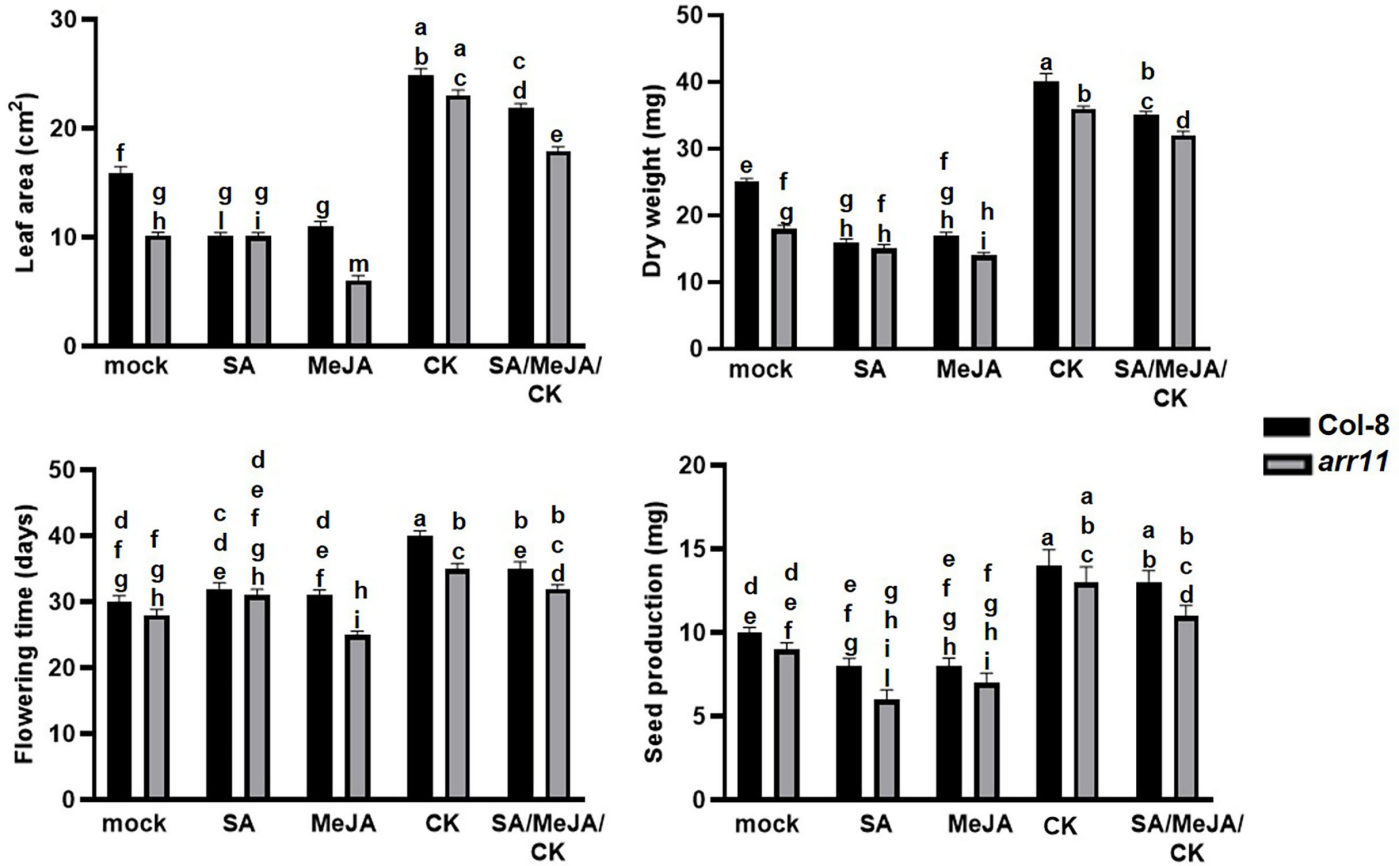
Fitness parameters of Arabidopsis Col-8 and *arr11* mutant. Leaf area (cm^2^), dry weight (mg), flowering time (days), and seed production (mg) were analyzed in Col-8 and *arr11* plants after single and triple hormone treatment. Error bars represent SEM. Letters indicates a statistically significant difference between treatments and genotypes (two-way ANOVA; *p<* 0.0001, n= 15). The experiments have been repeated three times with similar results.

To investigate the impact of ARR11 on plant fitness following SA/JA/CK treatment, leaf area, dry weight, day of flowering and seed production were measured in the *arr11* mutant and Col-8, after single and combined treatments with SA, MeJA, CK (**Figure 1**). A general reduction of fitness parameters, although not always statistically significant, was observed after SA and JA treatments in both Col-8 and *arr11*, compared to mock. Besides, CK was able to significantly increase leaf area, dry weight, flowering time and seed production in both Col-8 and *arr11* mutant, compared to mock. This positive effect was observed also after SA/MeJA/CK treatment, although slightly reduced. However, in general, the data did not show a significant difference between Col-8 and *arr11* across all parameters with mock or hormone treatment.

**Figure 2:**
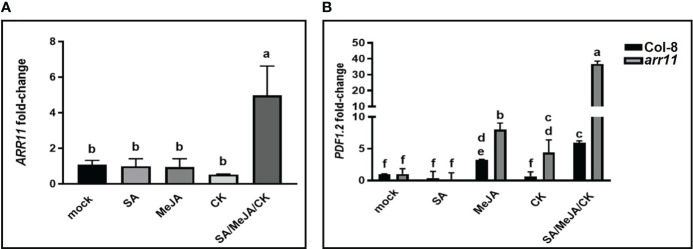
Gene expression analysis after hormone treatments. qRT-PCR analysis of *ARR11* **(A)** in leaves of Col-8 and of *PDF1.2* **(B)** in leaves of Col-8 and *arr11*, that were treated with a mock solution or with SA, MeJA, CK, SA/MeJA/CK. Fold change is relative to the expression in mock-treated plants and normalized to the reference gene *PP2AA3.* Gene expression analyses was performed 24 h after hormone treatment of 5-week-old plants. Letters indicates statistically significant differences between treatments and mock. One-way ANOVA, *p<* 0.05 **(A)**; two-way ANOVA, *p<* 0.0001 **(B)**.

We further investigated the involvement of ARR11 in the plant’s response to hormone treatments at molecular level. Firstly, *ARR11* gene expression was analyzed in Col-8 after treatment with SA, MeJA, CK alone as well as in combination. As shown in **Figure 2A**, while single hormone treatment had no effect on *ARR11* expression, SA/MeJA/CK treatment had a significant synergistic effect on *ARR11* expression. This result highlights a great impact of the triple hormone treatment on *ARR11* expression. Furthermore, since the JA pathway was perturbed in the *arr11* mutant (Proietti et al., 2018), we wondered if the SA/MeJA/CK treatment could also impact it. Thus, the expression of the JA marker gene *PDF1.2* was investigated (**Figure 2B**). MeJA was able to increase *PDF1.2* expression in both Col-8 and *arr11*, although more efficiently in the mutant. After CK treatment, the marker significantly increased in *arr11* mutant only. The triple hormone treatment had a synergistic effect on *PDF1.2* expression in both Col-8 and *arr11* mutant, compared to the single hormone treatment. This effect was definitely more pronounced in *arr11* compared to Col-8.

**Figure 3:**
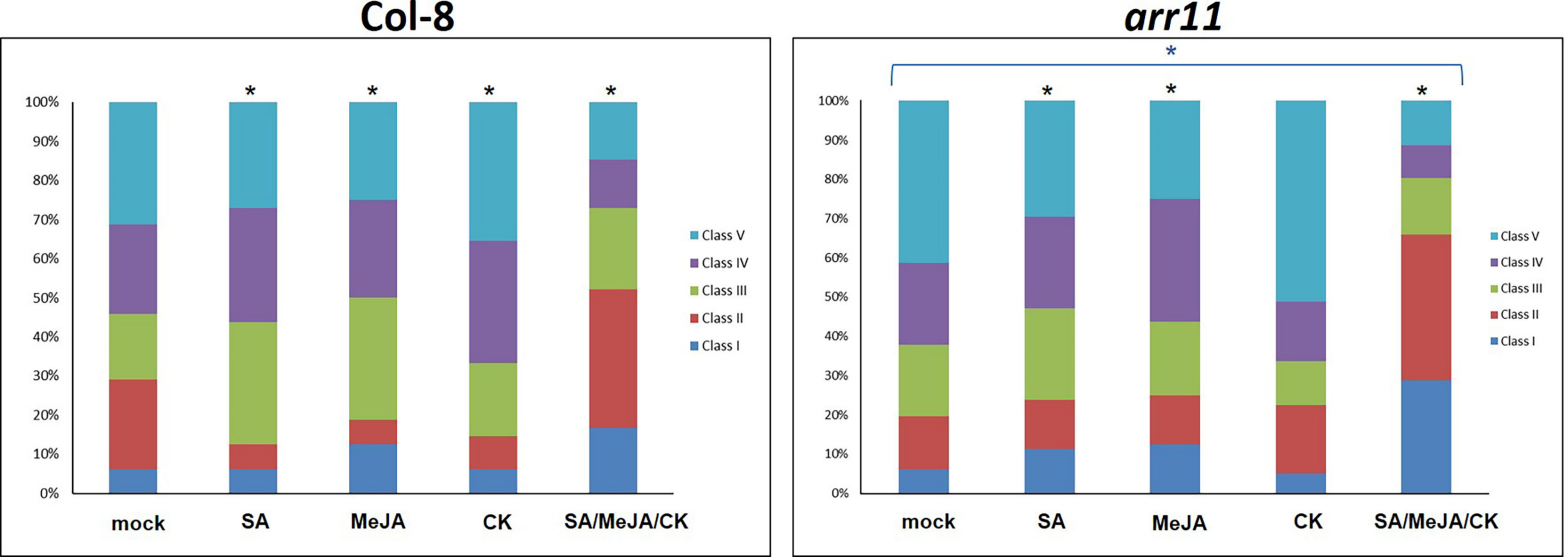
*B cinerea* bioassay in Col-8 and *arr11*. Distribution of disease symptoms of leaves of Col-8 and *arr11*, 3 days after inoculation with *B cinerea*. The bars indicate the frequency distribution of disease symptoms. Disease rating is expressed in 5 classes: I, from no necrotic lesion visible to lesion smaller than 3 mm; II, lesion between 4 mm to 1 cm; III, lesion bigger than 1 cm; IV, lesion between 1 cm and 1.5 cm; V, necrotic leaf. A black asterisk above the bars indicates significant differences between mock and each treatment (χ^2^-test, p-value<0.05). The blue asterisk above the bracket indicates significant differences in response to any treatment between *arr11* and Col-8.

We then tested the effect of single and combined SA/MeJA/CK treatment on *B. cinerea* resistance in both Col-8 and *arr11* mutant. In the mock, *arr11* mutant showed more susceptibility to *B. cinerea* compared to Col-8 and this result is in agreement with Proietti et al., 2018. The effect of single hormone treatment was visible in both Col-8 and *ar11* mutant, although with different magnitude. The most interesting result is that the combination SA/MeJA/CK led to an increased resistance to *B. cinerea*, compared to single treatments and to mock, in both Col-8 and *arr11* mutant (**Figure 3**). Noteworthy, under triple treatment, this resistance was strongly enhanced in *arr11* mutant compared to Col-8. The increased resistance to *B. cinerea* could be supported by the higher expression of *PDF1.2* in this sample (**Figure 2B**).

**Figure 4:**
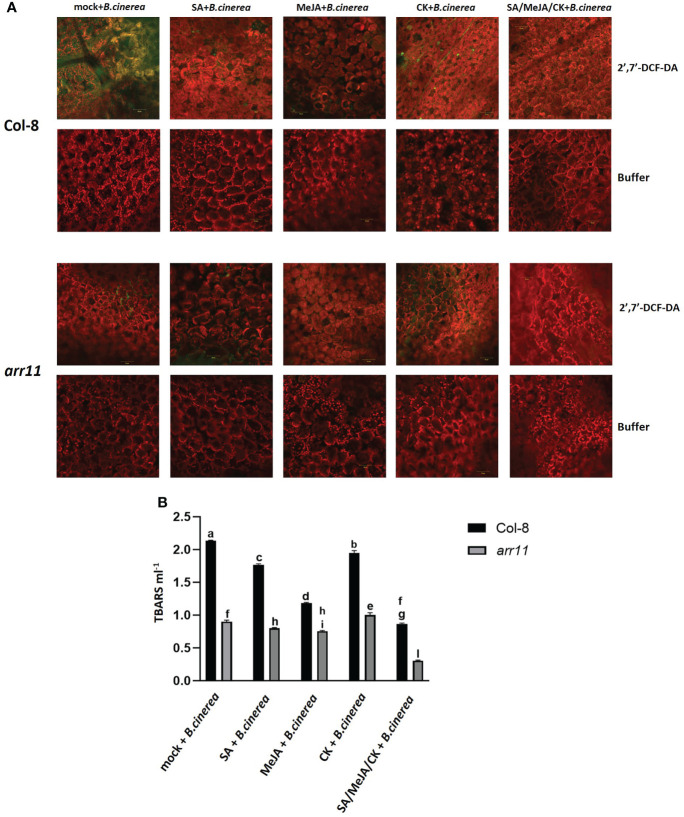
Detection of ROS and lipid peroxidation in Arabidopsis Col-8 and *arr11* leaves. **(A)** Detection of ROS on Col-8 and *arr11* leaves was carried out by using 2’,7’-DCFH_2_-DA or buffer (negative technical control). Fluorescence was observed under an LSM 710 confocal microscope with Plan Neofluar 20/1.30 objective. Laser excitations line was used, i.e., 488 for probe detection (green) and 561 nm for chlorophyll autofluorescence (red). Bar corresponds to 50 µm. The representative merged images are shown. **(B)** The level of TBARS was used to assess lipid peroxidation measuring the absorbance of MDA–TBA complex at 532 and 600 nm. Letters indicate statistically significant difference between different treatments and genotypes (two-way ANOVA, Tukey’s test p-value< 0.0001). Error bars represent means ± SDs (n = 3).

In order to test if the triple treatment has an effect on reducing *B. cinerea*-induced oxidative stress, ROS as well as lipid peroxidation were analyzed in both Col-8 and *arr11*, after single and triple treatment (**Figure 4**). ROS were detected in five-week-old leaves, after incubation with 2,7-dichlorofluorescein diacetate (2,7-DCFH_2_-DA), a compound largely used as ROS-sensitive dyes (Proietti et al., 2019). This molecule diffuses through the plasma membrane into the cytoplasm and is deacetylated by intracellular esterase before being oxidized by ROS to produce the green, fluorescent dye 2’,7’-DCF. Originally, it was assumed that oxidation of DCFH_2_ to DCF was limited to H_2_O_2_, but recent data has demonstrated that other ROS such as hydroxyl radical, hydroperoxides, and peroxynitrite may also oxidize DCFH_2_, albeit with much lower sensitivity than H_2_O_2_ (Proietti et al., 2019). The green spots that mark the presence of ROS, seemed very abundant in Col-8 infected by *B. cinerea*, while they were strongly reduced after MeJA and the triple treatment. On the other hand, in *arr11* mutant the effect did not seem to be hormone dependent since ROS were already low in the mock (representative photos are in **Figure 4A** and **Supplementary Figure 1**) The effectiveness of SA/MeJA/CK treatment in reducing lipid peroxidation was tested by TBARS assay (**Figure 4B**). The assay involves the reaction of lipid peroxidation products, primarily malondialdehyde (MDA) with thiobarbituric acid (TBA), which leads to the formation of MDA-TBA_2_ adducts called TBARS. Triple hormone treatment was able to reduce *B. cinerea*-induced lipid peroxidation with more pronounced effect in the *arr11* mutant than Col-8.

## Discussion

Plant hormones play pivotal role in several aspects of plant life and their signaling pathways can cross-communicate, leading to an optimization of plant adaptive responses. In Arabidopsis, one of the best investigated cross-talk is between SA and JA, with several other hormones playing as modulator of the backbone (Aerts et al., 2020). Among the latter are CKs, which are primarily involved in plant growth and development but also in stress responses (Choi et al., 2010; Robert-Seilaniantz et al., 2011; Giron et al., 2013; O’Brien and Benková, 2013). CK signaling network involves a number of ARR, among which is ARR11, which also recently emerged as a new SA/JA crosstalk regulator (Argyros et al., 2008; Proietti et al., 2018). Here, we investigated the impact of the triple hormonal treatment with SA, JA and CK on plant growth and JA-mediated defense as well as the involvement of ARR11 in both physiological responses. We found that SA/JA/CK treatment is able to restore the negative impact of SA and JA on growth parameters and, most interestingly, in general ARR11 positively impact plant fitness, both under hormone treatment and in mock (**Figure 1**). This result corroborated previous findings in which the lack of type-B ARR family members was deleterious for plant fitness and development (Imamura et al., 2003). At molecular level, we showed that the triple hormone treatment increased *ARR11* transcripts, suggesting a need for boosting under the effect of SA/JA/CK (**Figure 2A**). Moreover, SA/JA/CK had a great impact on the JA-responsive marker gene *PDF1.2*, and when *ARR11* was defective, this effect was even more pronounced (**Figure 2B**). Noteworthy, JA and CK alone were already able to increase *PDF1.2* expression in *arr11* mutant compared to Col-8 (**Figure 2B**), and the enhanced outcome triggered by the triple treatment could be due by a synergistic effect of CK and JA. As recently reported in rice, CK treatment is able to boost JA signaling pathway (Zhang et al., 2022). Besides the role as negative regulator in SA/JA crosstalk (Proietti et al., 2018), our results suggest that ARR11 could be a negative regulator of the JA signaling pathway under the effect of combined SA/JA/CK treatment. The JA-pathway is well known to be activated against the necrotrophic pathogens, such as *B. cinerea* (Pieterse et al., 2012). We found that SA/JA/CK increased the resistance against *B. cinerea* and this effect was even stronger when *ARR11* was defective (**Figure 3**), suggesting that ARR11 could play a role as negative regulator of the resistance against *B. cinerea* in these conditions. Furthermore, it is well known that under biotic stress plants increases ROS formation (Huang et al., 2019). As reported in Zwack et al., 2016, ARR11 is downregulated in response to oxidative stress, allowing improved stress tolerance. In our work, we observed that SA/JA/CK treatment was able to mitigate ROS generation, especially in Col-8. Lipid peroxidation induced by *B. cinerea* was also reduced by the triple hormone treatment and this effect was even more pronounced when *ARR11* was defective (**Figures 4A, B**).

In conclusion, taken together our results proved the importance of ARR11 in plant defense related processes. Future research will be performed to undisclosed the mechanism of action of ARR11 in multiple hormone pathways, as well as in response to plant pathogens.

## Data availability statement

The raw data supporting the conclusions of this article will be made available by the authors, without undue reservation.

## Author contributions

SP and CC designed the study. SP, GF, LB, and EB conducted the experiments. GF and LB analyzed the data. SP and CC wrote the manuscript. All authors contributed to the article and approved the submitted version.

## Funding

The authors acknowledge the PRIN grant (PROSPECT 2017JLN833_005) by Ministero dell’Istruzione, dell’Università e della Ricerca.

## Conflict of interest

The authors declare that the research was conducted in the absence of any commercial or financial relationships that could be construed as a potential conflict of interest.

## Publisher’s note

All claims expressed in this article are solely those of the authors and do not necessarily represent those of their affiliated organizations, or those of the publisher, the editors and the reviewers. Any product that may be evaluated in this article, or claim that may be made by its manufacturer, is not guaranteed or endorsed by the publisher.

## Supplementary material

Supplementary Figure 1 Detection and quantification of ROS, as detected in . **(A)** Detection of ROS on Col-8 and *arr11* leaves was carried out by using 2’,7’-DCFH_2_-DA or buffer (negative technical control). Fluorescence was observed under an LSM 710 confocal microscope with Plan Neofluar 20/1.30 objective. Green fluorescence only is showed. Bar corresponds to 50 µm. **(B)**. Quantification of green fluorescence detected by using 2’,7’-DCFH_2_-DA in Col-8 and *arr11* leaves, based on images of panel A. Heatmap of integrated density that sums all the pixels within the analyzed area giving a total value is shown. Quantification has been performed by Image J, version 1.53s.
