# Supplementary figures and images for: Plant defense: ARR11 response regulator as a potential player in Arabidopsis
Source: Front Plant Sci. 2022 Sep 21;13:995178. doi: 10.3389/fpls.2022.995178 (PMC9533103; doi:10.3389/fpls.2022.995178)

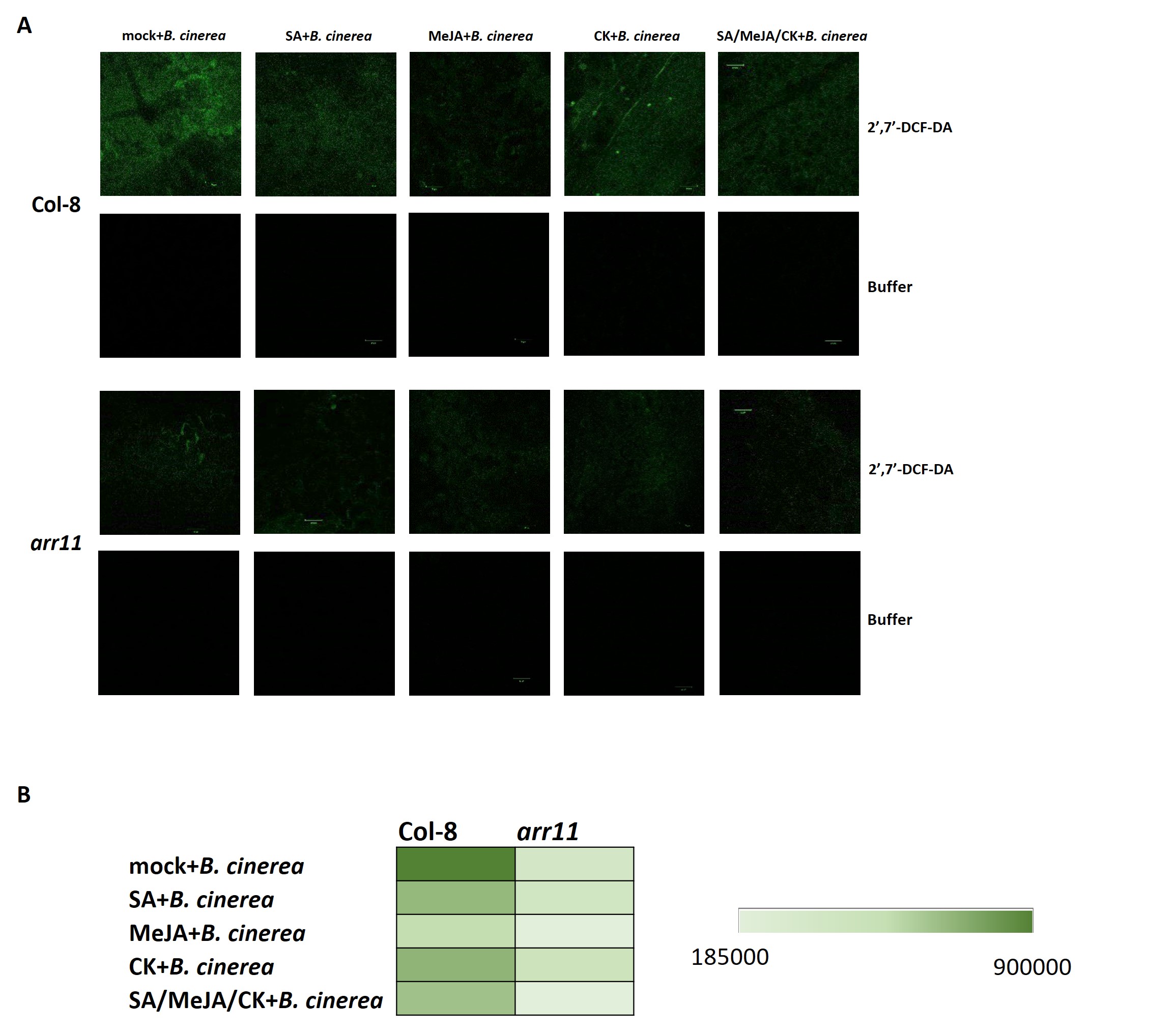

Supplement: Supplementary Figure 1 — Detection and quantification of ROS, as detected in . (A) Detection of ROS on Col-8 and arr11 leaves was carried out by using 2’,7’-DCFH2-DA or buffer (negative technical control). Fluorescence was observed under an LSM 710 confocal microscope with Plan Neofluar 20/1.30 objective. Green fluorescence only is showed. Bar corresponds to 50 µm. (B). Quantification of green fluorescence detected by using 2’,7’-DCFH2-DA in Col-8 and arr11 leaves, based on images of panel A. Heatmap of integrated density that sums all the pixels within the analyzed area giving a total value is shown. Quantification has been performed by Image J, version 1.53s. [file Image_1.jpeg]
